# TEDRAS II - transesophageal echocardiography as dysphagia risk in the acute phase of stroke—protocol for a prospective pilot observational trial

**DOI:** 10.1186/s13063-025-09065-5

**Published:** 2025-10-27

**Authors:** Samra Hamzic, Martin Juenemann, Tobias Braun, Kerstin Piayda, Pascal Bauer, Samuel Sossalla, Tibo Gerriets, Hassan Khilan, Marius Butz, Patrick Schramm, Omar Alhaj Omar

**Affiliations:** 1 Department of Neurology, University Hospital Giessen and Marburg, Klinikstr. 33, Giessen, 35392 Germany; 2 Department of Cardiology, University Hospital Giessen and Marburg, Klinikstr. 33, Giessen, Germany; 3 Swiss University of Speech and Language Sciences - hlo, Bahnhofsplatz 2, St. Gallen, 9000 Switzerland; 4 Kerckhoff-Clinic, Heart & Brain Research Group, Benekestr. 2-8, Bad Nauheim, 61231 Germany; 5 Die Neurologen, Private Neurology Practice, Frankfurter Str. 34, Bad Nauheim, 61231 Germany

**Keywords:** Acute stroke, Dysphagia, Swallowing, FEES, TEE

## Abstract

**Background:**

Dysphagia is as a common consequence of cerebral infarction affecting approximately 50% of stroke patients. It often results in aspiration pneumonia, malnutrition and dehydration. Dysphagia is also seen after mechanical manipulation of the larynx, for example after prolonged intubation and after the perioperative use of transesophageal echocardiography (TEE). TEE is a semi-invasive procedure assessing the function of the intra-atrial septum and heart valves, yielding more detailed results compared to transthoracic echocardiography (1). It has become a routine in the diagnostic work-up of stroke. The study *Transoesophageal echocardiography: dysphagia risk in the acute phase after stroke* (*TEDRAS study*) was the first to investigate the extent of dysphagia risk after TEE in acute stroke patients. The trial findings supported the hypothesis that dysphagia severity worsens following TEE. This follow-up trial is designed to address the limitations of the first TEDRAS study, focusing on patients with ischaemic stroke and transient ischemic attack (TIA).

**Methods:**

The study is a prospective, single-blinded, controlled pilot trial, designed in accordance with the SPIRIT reporting guidelines. The study protocol outlines that both FEES and TEE will be performed on patients with acute ischaemic stroke or transient ischemic attack (TIA) who provide informed consent to participate. Specifically, the following parameters are to be analyzed in both groups: (1) The impact of the type and method of anaesthesia administration (intravenous vs. local anesthetic spray application) during TEE on swallowing function in both cohorts; (2) The effect of the duration of the TEE procedure on swallowing function in both cohorts; (3) The experience level of the TEE examiner; (4) The interrater reliability of the FEES assessment. The trial will be conducted at the University Medical Centre of the Justus-Liebig-University in Giessen (UKGM) through collaboration between the Departments of Neurology and Cardiology of the UKGM.

**Discussion:**

This protocol presents a prospective pilot single-blinded controlled study designed to assess further risks of dysphagia following TEE. The trial aims to address several outcomes, specifically the impact of anaesthesia and the duration of TEE on swallowing, which will be correlated with the severity of dysphagia after TEE.

**Trial registration:**

ClinTrial.gov Identifier NCT04302883.

URL: https://clinicaltrials.gov/study/NCT06195501?term=TEDRAS&rank=1

Registered June 2024.

## Administrative information

**Table:** 

Title and structured summary	(1a) TEDRAS II - Transesophageal Echocardiography as Dysphagia Risk in the Acute phase of Stroke - protocol for a prospective pilot observational trial (1b) This pilot follow-up trial is designed to address the impact of the duration of TEE on swallowing in acute stroke patients and patients with transient ischemic attack, the type of anesthesia as well as the inter- and intrarater reliability for FEES. We will specifically analyse the following parameters in both groups: 1) The impact of the type and method of anaesthesia administration (intravenous vs. local anesthetic spray application) during TEE on swallowing function in both cohorts. 2) The effect of the duration of the TEE procedure on swallowing function in both cohorts. 3) The experience level of the TEE examiner. 4) The interrater reliability of the FEES assessment. The study is a pilot prospective, single-blinded, controlled trial, designed in accordance with the SPIRIT reporting guidelines. The study protocol outlines that both FEES and TEE will be performed on patients who provide informed consent to participate. Patients with acute ischemic stroke or TIA that provided their consent to participate are included in the trial.
Protocol version	(2) Protocol version #1, TEDRAS 2 June 2024
Roles and responsibilities	(2) **Names:** Samra Hamzic^1, 3^ Martin Juenemann^1^ Tobias Braun^1^ Kerstin Piayda^2^ Pascal Bauer^2^ Samuel Sossalla^2^ Tibo Gerriets^1,5^ Hassan Khilan^1^ Marius Butz^4^ Patrick Schramm^1^ Omar Alhaj Omar^1^ **Affiliations:** ^1^University Hospital Giessen and Marburg, Campus Giessen, Department of Neurology, Giessen, Germany ^2^University Hospital Giessen and Marburg, Campus Giessen, Department of Cardiology, Giessen, Germany ^3^Swiss University of Speech Therapy - hlo, St. Gallen, Switzerland ^4^Kerckhoff-Clinic, Heart & Brain Research Group, Bad Nauheim, Germany ^5^Die Neurologen, Private Neurology Practice, Bad Nauheim, Germany **Roles:** Principal investigator: Samra Hamzic Key clinical investigators: Omar Alhaj Omar, Martin Juenemann, Patrick Schramm, Hassan Khilan, Kerstin Piayda, Pascal Bauer, Samuel Sossalla Independent experts: Tibo Gerriets, Tobias Braun, Marius Butz
	**(3b and 3c)** There is no external or internal funding for this trial. It is conducted exclusively within the clinical routine of the Departments of Neurology and Cardiology at the University Hospital Giessen and Marburg, Campus Giessen. This is an investigator-initiated trial. Since there are no trial funders, the investigators retain full control over all study related decisions.
Protocol and statistical analysis plan	**(5)** Protocol and statistical analysis plan can be accessed under: ClinTrial.gov Identifier NCT04302883. URL: https://clinicaltrials.gov/study/NCT06195501?term=TEDRAS&rank=1
Data sharing	**(6)** Patient data and the case report fomr (CRF) are stored on the internal password protected server of the study center.

## Background

Dysphagia is as a common consequence of cerebral infarction, affecting at least 50% of stroke patients [1]. It often results in aspiration pneumonia, malnutrition and dehydration which are considered the most important secondary complication on the stroke unit [2]. Up to 50% of stroke patients suffer dysphagia 6 months from experiencing an ischaemic stroke [3]. Additionally, dysphagia causes numerous other secondary complications [4–6], contributing to substantial healthcare cost [7–9]. 

Dysphagia involves a complex pattern of swallowing dysfunction; the aetiology is heterogeneous [10]. The oropharynx is a highly sensitive structure transmitting information about the composition of the bolus to the cortex. Proper function of the tongue and muscles of the mouth floor is crucial for bolus formation and expulsion. In the hypopharynx, pressure dynamics and the contraction force of the pharyngeal muscles are essential for bolus expulsion into the oesophagus [11]. Damage to the muscles or nerves in this area can result in serious swallowing dysfunctions, increasing the risk of aspiration.

The complex protective mechanism of the upper airway during swallowing must operate precisely to protect the airway as the bolus is propelled into the oesophagus. Injuries or surgical interventions involving the hypopharynx and larynx are particularly critical. Dysphagia is commonly seen after prolonged intubation [12–14] and has been reported after the perioperative use of transoesophageal echocardiography (TEE) [15].

TEE, a semi-invasive procedure, provides enhanced visualization of the heart’s structure and function, particularly for assessing the intra-atrial septum and heart valves, often yielding more detailed results compared to transthoracic echocardiography [16]. It plays a crucial role in the diagnostic work-up of ischaemic stroke, particularly in identifying cardioembolic sources such as left atrial appendage thrombus (LAAT) and spontaneous echo contrast (SEC). Recent studies have demonstrated that TEE provides prognostically significant findings even in anticoagulated patients with atrial fibrillation (AF), where the CLOTS-AF score (*C*reatinine levels > 1.5 mg/dL (2 points), *L*VEF < 50% (2 points), LAVI > 34 mL/m^2^ (indicatingng *O*verload, 1 point), *T*APSE < 17 mm (2 points), history of prior **s**troke (3 points) and presence of *AF* rhythm (2 points). The total score can range from 0 to 12 points and has shown superior predictive accuracy for LAAT compared to conventional risk scores [17–20]. Moreover, TEE has revealed a substantial prevalence of thrombus in stroke patients without documented AF, highlighting the importance of structural cardiac abnormalities as embolic sources. These findings underscore the value of TEE in refining risk stratification and guiding secondary stroke prevention, especially in patients with cryptogenic or embolic strokes of undetermined source (ESUS).

While it is generally considered a minimal invasive and safe procedure, complications such as mucosal injuries, bleeding in the upper gastrointestinal tract or aspiration pneumonia can still occur. In relation to swallowing, injuries to the oesophagus, larynx or pharynx may contribute to dysphagia [21–23]. The duration of the TEE procedure may also influence these risks [24].

Professional and effective dysphagia management and successful rehabilitation after stroke depend on targeted and standardized imaging diagnostics to assess both symptoms and underlying pathomechanisms. In Germany, Flexible Endoscopic Evaluation of Swallowing (FEES) has become the most widely used imaging tool for diagnosing dysphagia [25]. Since the introduction of the FEES curriculum by the German Society of Neurology (DGN) and the German Stroke Association (DSG) in 2014 [26], this imaging method has been implemented in 70% of stroke units across the country [25].

The procedure is performed by inserting a flexible videoendoscope, approximately 3 mm in diameter, through the lower nasal passage into the hypopharynx. FEES is video recorded, with playback capabilities of 25–50 frames per second, allowing precise identification of key symptoms and pathomechanisms. It can be performed at the patient’s bedside, even in challenging conditions, and repeated as often as required. This diagnostic tool is considered low-risk: a trial of 300 acute stroke patients reported no significant changes in cardiorespiratory parameters, no laryngospasm, vasovagal reaction, altered consciousness, or symptomatic bradycardia or tachycardia during the procedure [27].

The study *Transoesophageal echocardiography: dysphagia risk in the acute phase after stroke* (*TEDRAS study*) approved by the ethics committee of the Justus Liebig University in Giessen (AZ.: 223/12; ClinTrial.gov Identifier NCT04302883) [28] was the first to investigate the extent to which TEE increases the risk of dysphagia in acute stroke patients. In this trial, patients with an acute ischaemic stroke were randomized into an intervention or a control group. The intervention group underwent FEES on 3 consecutive days, with the TEE taking place on the second day before the FEES. The control group also received FEES over 3 days, but TEE was performed only after the final FEES. The final findings supported the hypothesis that dysphagia severity worsens following TEE in the intervention group [28].

### Objectives

Building on the results of the initial TEDRAS trial, this follow-up trial is designed to address the limitations of the first TEDRAS study that were not sufficiently explored: The impact of the duration of TEE on swallowing, the type on anaesthesia as well as the interrater reliability for FEES. In the follow-up TEDRAS trial we will be focusing on patients with ischaemic stroke and transient ischemic attack (TIA) specifically analyzing the following parameters in both groups:

1. The impact of the type and method of anaesthesia administration (intravenous vs. local anesthetic spray application) during TEE on swallowing function in both cohorts.
2. The effect of the duration of the TEE procedure on swallowing function in both cohorts.
3. The experience level of the TEE examiner.
4. The interrater reliability of FEES.

## Methods

**Fig. 1:**
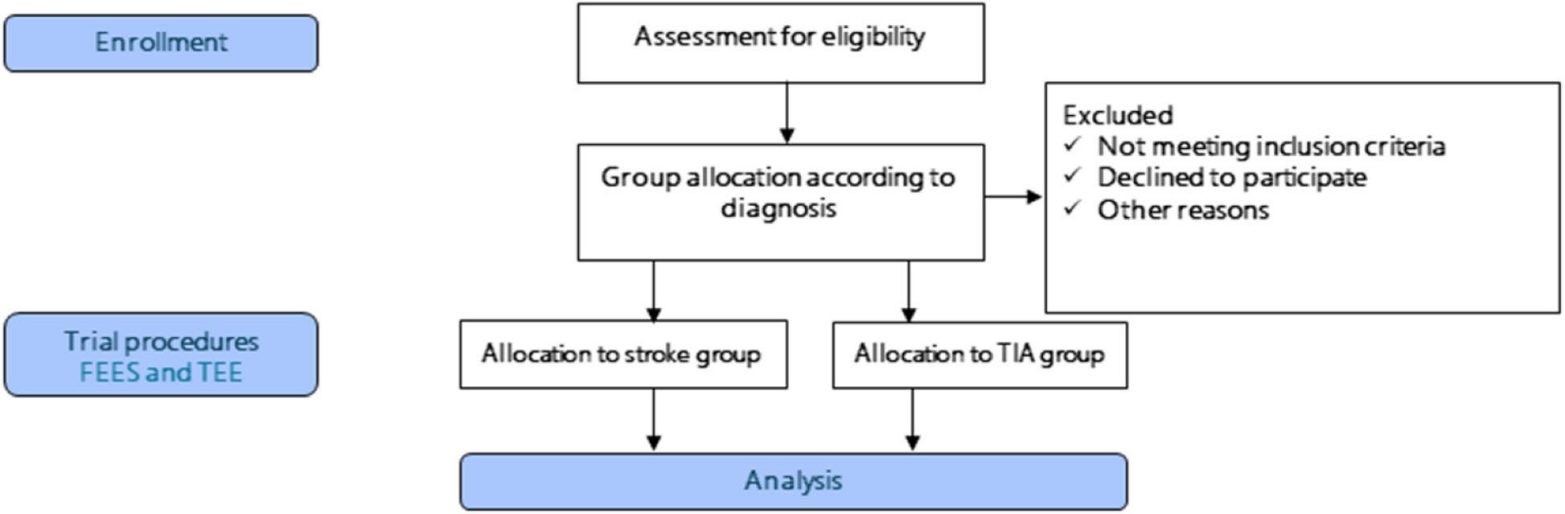
Patient recruitment flow diagram

The study is a prospective, single-blinded, controlled trial, designed in accordance with the SPIRIT reporting guidelines [29]. The study protocol outlines that both FEES and TEE will be performed on patients who provide informed consent to participate. Patients with acute ischaemic stroke or transient ischemic attack (TIA) that provided their consent to participate are included in the trial. The patient recruitment flow diagram is presented in Fig. 1.

### Study setting

The trial will be conducted at the University Medical Centre of the Justus-Liebig-University in Giessen (UKGM) through collaboration between the Departments of Neurology and Cardiology of the UKGM.

### Eligibility criteria

A total of 50 patients will be recruited from the UKGM stroke unit, comprising 25 with ischaemic stroke and 25 with TIA.

The inclusion criteria are (1) ischaemic stroke occurring no more than 7 days prior, confirmed by cranial computed tomography (cCT) or magnetic resonance imaging (MRI); (2) for TIA: presence of a perfusion deficit in cCT or clinical diagnosis of TIA by a physician with focal neurological deficits still persisting at the time of presentation; (3) indication for TEE in both groups; (4) written informed consent to participate.

Exclusion criteria include (1) pre-existing neurogenic dysphagia; (2) prior surgery or chemoradiotherapy for head and neck tumours; (3) cervical spine surgery; (4) thyroid gland surgery; (5) aphasia; (6) acute delirium; (7) lack of compliance; (8) lack of alertness; (9) dementia; (10) existing legal guardianship.

TEE will be performed by cardiologists from the Department of Cardiology at UKGM. FEES will be conducted by neurologists and/or speech and language therapists (SLT) from the Department of Neurology at UKGM.

Trial participants or authorized surrogates will be informed in detail and comprehensibly both in a written consent form as well as in a personal interview by the medical investigator from the Department of Neurology about the treatment method to be tested and the comparative method as well as about the nature, significance, risks and scope of the clinical trial.

###  Interventions

Professional and effective dysphagia management and successful rehabilitation after stroke depend on targeted and standardized imaging diagnostics to assess both symptoms and underlying pathomechanisms. In Germany, FEES has become the most widely used imaging tool for diagnostics of dysphagia [25]. Based on evidence-based dysphagia scores, investigators are able to determine both the severity of dysphagic symptoms and adequate therapy and diet [30–34].

TEE, a semi-invasive procedure, provides enhanced visualization of the heart’s structure and function, particularly for assessing the intra-atrial septum and heart valves, often yielding more detailed results compared to transthoracic echocardiography [16]. Over the past few decades, TEE has become a routine examination in the diagnostic work-up of stroke. While it is generally considered a minimal invasive and safe procedure, complications such as mucosal injuries, bleeding in the upper gastrointestinal tract or aspiration pneumonia can still occur. In relation to swallowing, injuries to the oesophagus, larynx or pharynx as well as the duration of TEE may contribute to dysphagia [21–24].

### Intervention description

#### Equipment and procedures for FEES

The FEES procedure at UKGM utilizes the Rehder/Partner Swallowing Workstation (Hamburg, Germany), equipped with a Karl Storz 3.6-mm HD videoendoscope and camera system. The endoscopic sequences are recorded using a digital picture archiving and communication system (Rehder/Partner), with an image capture rate of 35 frames per second and a resolution of 1021 × 1021 pixels.

#### Pre-procedural preparation

Before the FEES, nasal decongestion is achieved by administering anti-congestive nose drops. Additionally, nasal mucosa and the nostrils are anesthetized using topically applied lidocaine, delivered via cotton swabs.

#### FEES protocol

**Fig. 2:**
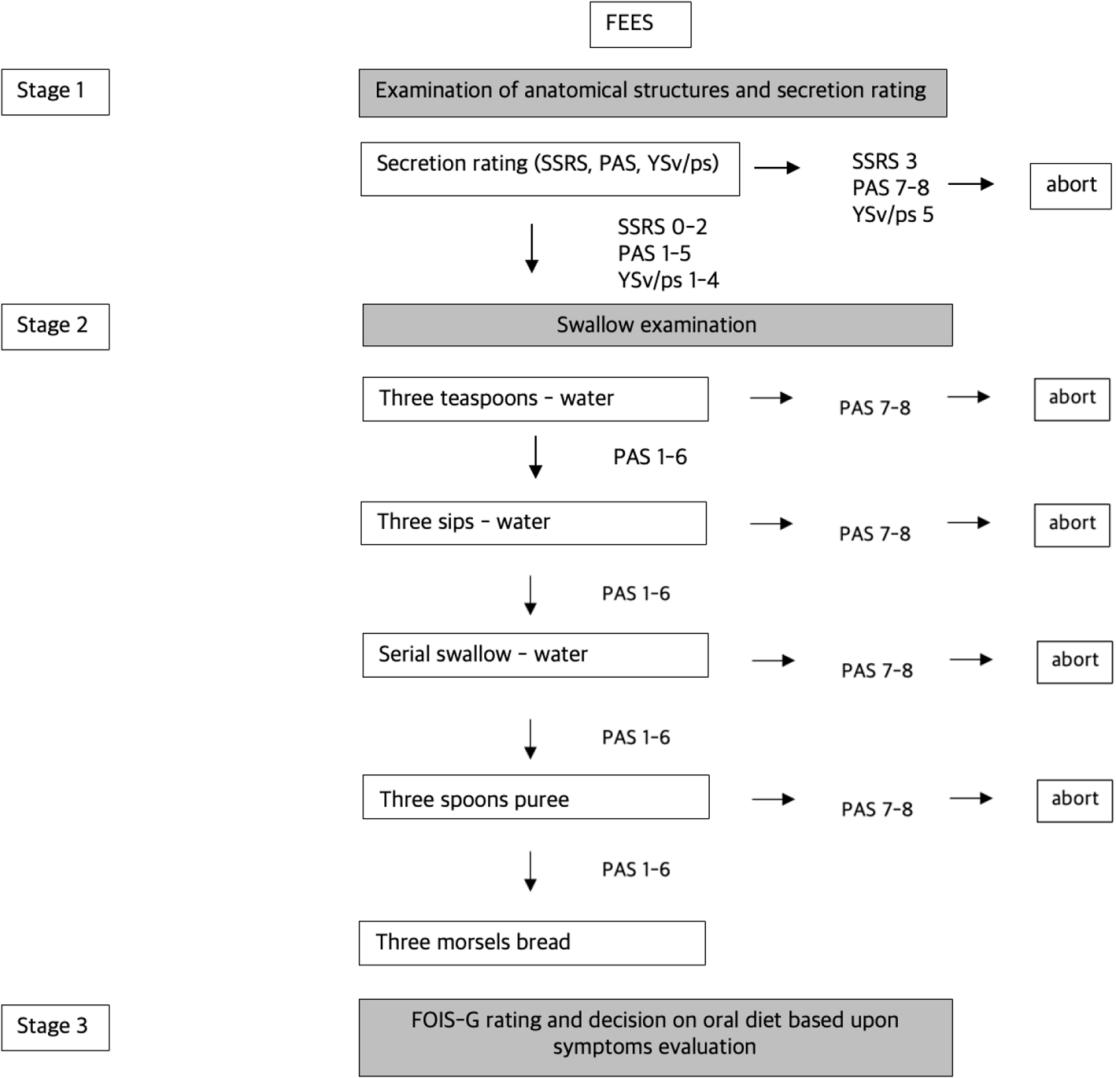
UKGM standardized FEES examination protocol. Abbreviations: FEES = Flexible Endoscopic Evaluation of Swallowing; SSRS = Secretion Severity Rating Scale; PAS = Penetration-Aspiration-Scale; YSv = Yale Pharyngeal Residue Severity Rating Scale for Valleculae; YSps = Yale Pharyngeal Residue Severity Rating Scale for Piriform Sinus; FOIS-G = Functional Oral Intake Scale (German Version)

FEES is conducted according to a standardized examination protocol of the UKGM (Fig. 2). FEES is performed either by a neurologist or a speech and language therapist (SLT) both board certified with the FEES Certificate of the German Society of Neurology. Initially, a preliminary assessment of the patient’s management of saliva is conducted. Subsequently, the patient is instructed to swallow various test boluses (three teaspoons of water; three sips of water; three teaspoons of applesauce; and three morsels of crispbread). 

#### Dysphagia severity assessment via FEES

Dysphagia severity is evaluated using following dysphagia scores:

1. Secretion Severity Rating Scale (SSRS): this 4-point scale assesses the patient’s ability to manage saliva and secretions, which correlates with the risk of aspiration pneumonia. Higher scores on this scale indicate a greater risk of aspiration pneumonia and respectively more severe dysphagic symptoms [32].
2. The Penetration-Aspiration Scale (PAS): This 8-point scale evaluates the extent of penetration and aspiration of saliva, liquids and food. It also describes the patient’s sensory response to penetration or aspiration and their ability to expectorate the penetrated or aspirated material [30]. Higher scores on this scale indicate more severe dysphagic symptoms with PAS-scores 6–8 indicating different severities of aspiration.
3. Yale Pharyngeal Residue Severity Scale (YS): A 5-point scale that measures the severity of residue in the hypopharynx after swallowing saliva, food and liquids [31]. Higher scores on this scale indicate more severe dysphagic symptoms.
4. Functional Oral Intake Scale: Following each FEES assessment, the patient’s ability to orally intake liquids and food is measured by the 7-point Functional Oral Intake Scale (FOIS-G, German version [33]). Lower scores on this scale indicate a deterioration in oral intake ability, with levels 1–3 corresponding to various levels of non-oral feeding and levels 4–7 corresponding to different levels of oral feeding [33, 34].

#### TEE

The Department of Cardiology at the University Medical Centre of the Justus-Liebig-University in Giessen conducts the TEE using a Philips EPIC 7 C ultrasound workstation and Philips Type X7 and X8 probe. For the procedure, anaesthesia is administered either intravenously with propofol or as a combination of intravenous propofol and local pharyngeal anaesthesia using lidocaine spray. Patients are required to refrain oral intake of food and liquids for at least 6 h prior to the TEE. This restriction continues for 30 min and up to 1 h after the TEE to ensure and minimize complications of potential post TEE dysphagia.

#### Criteria for discontinuing or modifying allocated interventions

The criteria for participant dropout include the following: (1) withdrawal of informed consent by the participant or the authorised surrogate; (2) suicidal behaviour during the trial; (3) occurrence of serious adverse events during the trial; (4) withdrawal deemed necessary for safety or effectiveness; (5) poor compliance with the study protocol; (6) significant violations of the trial protocol by the participant; (7) pregnancy during the study; and (8) inability to maintain contact (lost to follow-up).

#### Criteria for terminating FEES examination

FEES is aborted for any tested substance consistency in case of aspiration of saliva with PAS score > 6 or aspiration of liquid, puree or solid boli during the examination with PAS score > 6.

This protocol ensures a systematic approach to the evaluation of dysphagia and helps assess the risk of aspiration and functional swallowing outcomes in patients.

#### Criteria for terminating TEE examination

TEE examination will be terminated in case of overt gastrointestinal bleeding caused by the examination probe, severe hemodynamic and / or respiratory instability of the patient.

#### Strategies to improve adherence to interventions and auditing

The Project Management group consists of the principal investigator (PI), the study coordinator (SC) and specific personell (medical doctors (MDs) and SLTs).

The project will be managed and coordinated under the guidance of the principal investigator (PI). MDs from the Department of Neurology will be assigned to handle subject enrollment and clinical evaluation, whereas testing will be conducted by an MD and an SLT from the Department of Neurology and by an MD from the Department of Cardiology to ensure complication-free trial progress and maintain project quality. Inter-departmental coordination and communication meetings will be overseen by a project coordinator. The PI will oversee review progress, identify implementation issues and develop solutions in a quarterly group meeting. Quality control personnel and data verification personnel will be appointed to the trial team implementing a quality control plan.

The aim of the quality control plan is to align with the technical framework, ensuring adherence to procedures and systems for data collection, entry and database management. After initiation, regular quality and technical auditing reviews will be conducted by the ethics commitee, prompt feedback given and adjustments made to maintain high execution standards.

#### Protocol amendments

Changes made to the protocol that was appraised positively by the ethics committee must be positively reappraised and approved if the changes

- are such that they may affect the subjects’ safety;
- result in further data collection that necessitates changes to the patient information and/or informed consent form;
- affect the interpretation of the scientific documents upon which the trial is based or the significance of the results of the trial;
- significantly affect the leadership or conduct of the trial.

In order to ensure most comparable conditions during trial conduct and in the interest of valid statistical analyses, the lead coordinating investigator or any other person involved in the trial conduct may not alter the study conditions agreed upon and set out in this protocol.

Amendments should be made only in exceptional cases. Any amendment must be set out in writing, at the same time giving the reasons, and signed by all parties concerned. The amendment then becomes part of the study protocol.

Amendments which might have an impact on the well-being of the subject (major amendments) such as the use of additional invasive procedures require an additional approval by the ethics commitee. In addition, a further informed consent form must be signed by all trial subjects enrolled in the trial who might be affected by the amendment. In case of substantial changes, new approvals of the leading ethics committee are required before the changes become effective. Minor changes will only be submitted to the ethics committee in a written form.

#### Relevant concomitant care permitted or prohibited during the trial

Any pharmacological treatment that contradicts the acute treatment of stroke is prohibited. SLT interventions (diagnostic and therapeutic) that ensure an aspiration free dysphagia management are possible during the trial period. At the conclusion of the trial, participants requiring additional treatment of dysphagia will be supported in securing appropriate follow-up care in an outpatient setting or a rehabilitation facility.

## Outcomes

### Primary outcome

The primary outcome will assess the impact of the type and form of anaesthesia (intravenous vs. local) administered during TEE on swallowing in patients with ischaemic stroke and TIA, based on the aforementioned dysphagia scores [30–34].

### Secondary outcomes

1. Comparison of TEE duration between patients with and without dysphagia and in cases of ischaemic stroke and TIA;
2. Correlation between TEE duration and the degree of deterioration in FOIS-G and the experience of the TEE examiner in patients with ischaemic stroke and TIA;
3. Correlation between TEE duration and TEE examiner’s experience in patients with ischaemic stroke and TIA;
4. Interrater reliability in the evaluation of FEES.

### Sample size estimation

Given the lack of existing data on the influence of anaesthesia type and form during TEE on swallowing in ischaemic stroke and TIA patients, this study is designed as a pilot trial. A total of 50 participants will be enrolled, with 25 patients in each group (stroke vs. TIA). The primary aim of this exploratory phase is to gather preliminary data to estimate effect sizes. These estimates will then serve as the basis for a formal power calculation and definitive sample size planning for a subsequent full-scale study.

### Recruitment

Before the trial onset, all clinical staff at the recruitment site will be provided with information about the trial to share and discuss with potential participants. Therefore, all patients admitted to the stroke unit at UKGM in Giessen will be screened for eligibility to participate in the trial. If this approach does not yield enough participants, study-related advertisements will be displayed to aid in recruitment.

### Assignment of interventions: allocation

Since this is not a randomized trial the participants will be assigned to the aforementioned groups according to the diagnosis. The allocation sequence will be sequentially numbered by the allocating investigator MD and stored in a secure database only accessible by this person. The trial investigator responsible for enrollment and allocation will provide patients’s enrollment sequence number and allocate participants to respective groups after viewing the diagnosis. All enrolled patients will receive the same interventions in both groups.

### Blinding

This is a single-blinded trial. All evaluators of FEES intervention and the statisticians will be blinded for the allocation to the groups respectively the diagnosis of participants. For the unbiased evaluation of the FEES procedure, the FEES video sequences and all trial outcomes will be randomized and pseudonymized by an independent examiner. They will then be presented to a neurology specialist and an SLT, each with at least 5 years of experience treating stroke patients and certified in FEES by the German Society of Neurology and the European Society for Swallowing Disorders. Both the neurology specialist and the SLT will be blinded to group allocation and all previous results, and they will assign SSRS, PAS, YS and FOIS-G scores to all consistencies applied in the swallowing evaluation.

### Procedure for unblinding if needed

Unblinding is limited to critical situations, such as serious adverse events or life-threatening conditions requiring knowledge of the treatment for proper medical care. The decision to unblind must involve the PI with proper documentation of the justification and approval. In case of unbliding treatment, assignments will be accessed only when authorized by the PI. The unblinding procedure will comprise the verification of the participant’s identity and retrieving the allocation details by accessing a secure database. The information then will be shared with the medical team handling the participant. The full event will be recorded and documented comprising key details (participant ID, date, time, reason for unblinding and personnel involved). These records will be kept as part of the trial documentation. In order to limit the impact of unblinding on the study the unblinding will be restricted to the specific participant involved, ensuring no other participants, study staff or researchers are exposed to the allocation information. If required, ethics committee and any regulatory authorities will be informed Table 1.

**Table 1:** Participant timeline

**Interventions**		**Timepoint**				
		***Baseline***	***T1***	***Within 3–5 d after admission***	***T2***	***T3***
***Routine treatment***	Admission to stroke unit	x				
	NIHSS score	x				
	MRS score	x				
	BI score	x				
	CCT	x				
	Routine stroke treatment	x				
	Dysphagia assessment/FEES (if necessary)	x				
***Enrollment***	Inclusion/exclusion criteria	x				
	Informed consent	x				
	Group allocation according to diagnosis	x				
***Interventions***	FEES 1		x			
	TEE			x		
	FEES 2				x	
	FEES 3					x

### Data collection and management

We will collect demographic information at baseline from the digital patient file. Symptom evaluation of stroke or TIA will be conducted by a neurological MD. Evaluation of cCT will be performed and documented by a neuroradiologist within the routine work-up of stroke. Within the routine stroke treatment stroke relevant scores National Institutes of Health Stroke Scale (NIHSS), Modified Rankin Scale (MRS) and Barthel Index (BI) will be documented. NIHSS is a 15-item assessment tool designed to evaluate stroke severity. According to the latest guidelines from the National Stroke Foundation, it is endorsed as a reliable instrument for assessing stroke severity in emergency department settings [35]. MRS is a clinician-reported tool that assesses global disability on a 7-point scale, ranging from no symptoms and perfect health to death. It is the most commonly used outcome measure in acute stroke trials [36]. BI is a ten-item scale used to assess the ability to perform activities of daily living (ADLs), including tasks like feeding, bathing, managing continence, mobility and dressing. It has been shown to have strong internal consistency and validity when evaluating functional status in elderly patients after cerebrovascular events [37]. Swallowing assessment within 24 h after admission to the stroke unit is conducted by an SLT. The 70-ml water swallow test is administred to define aspirations risk [38]. FEES is conducted prior to enrollment to the trial if the swallowing assessment confirms aspiration risk and is needed to provide adaqute dysphagia management and diet recommendations. Upon the enrollment in the trial following dysphagia relevant FEES scores are documented: SSRS, PAS, YS and FOIS-G [30–33].

### Plans to promote participant retention and complete follow-up

N/a. All patients will receive the planned interventions within the routine stroke treatment according to the guidelines of the German Society of Neurology.

### Patient public involvement

No patient public involvement has been planned for this trial.

### Data management

Before starting participant enrollment, research staff will receive thorough training on how to complete case report forms (CRFs) and how to retrieve relevant information from the digital patient file. Thus, the procedural guidelines specified in the protocol are clarified with the goal of improving the consistency and reliability of the data. Throughout the trial, all data records and report will be accurately checked for completeness. Treatment changes, medications, withdrawals and missed assessment will be verified and recorded for every participant. The CRF will be thoroughly reviewed at the end of trial enrollment in order to correct all errors or omissions. Any necessary changes or comments will be signed and dated by the PI. Any issues discovered from discrepancies will be promptly resolved, either through additional data, corrections or clarifications from the PI. A data manager will perform a second review of the checked CRFs to identify any potential issues that may have been missed. This process will help with further corrections or additions as needed.

### Confidentiality

Trial participants will receive a unique code upon enrollment in the trial. The statisticians and the evaluating team will only have access to the patient code. No disclosure of real patient information will be available. All personal participants’ details will be stored in a secured database and accessible only to authorized members of the research team.

### Statistical methods

Patient-related data such as age, gender, diagnosis, relevant medication, TEE duration and application form of anaesthesia (local or intravenous) are evaluated descriptively with frequency data, mean values with standard deviations or medians with interquartile ranges.

### Definition of dysphagia

Dysphagia is determined with the following cut-off values of the dysphagia scores:

Secretion Severity Rating Scale for Saliva Management (SSRS): SSRS ≥ 1; Penetration Aspiration Scale (PAS): PAS ≥ 3; Yale Pharyngeal Residue Severity Rating Scale: YS ≥ 3; Functional Oral Intake Scale (FOIS-G): FOIS-G < 7. Differences in patient-related data between the two groups: dysphagia/non-dysphagia group and intravenous vs. oral anaesthesia group are tested using Pearson’s chi-square test, Fischer’s exact test, *t*-test for independent samples or the Mann–Whitney *U*-test. For the purpose of logistic regression analyses, we intend to apply a median split to dichotomize the SSRS, PAS, YS and FOIS-G scores and create a binary outcome variable.

Assumptions for a *t*-test for independent samples are tested with the Levent test for variance homogeneity and the Shapiro–Wilk test including visual inspection of thedistribution plots for the assessment of normal distribution. If there is no homogeneity of variance or normal distribution, the Mann–Whitney *U* test for group differences is used. Correlations are calculated using the Pearson product-moment correlation or the Spearman rank correlation.

The inter-rater reliability between two raters is determined using a linearly weighted Cohen’s kappa, as this takes into account matches that could have arisen by chance on the one hand and places more weight on high non-matches on the other [39]. Furthermore, the percentage agreement and the Spearman rank correlation are specified for the agreement between two raters. In the case of a calculation of inter-rater reliability with more than two raters, a general agreement of all raters including subtraction of agreements by chance is calculated using Fleiss kappa [39].

Effect sizes are given according to Cohen’s d with a 95% confidence interval. *p*-values are given for all statistical results. The criterion for statistical significance is set at *p* < 0.05. Conclusions about clinically, practically and scientifically relevant correlations or differences are not made solely on the basis of the defined statistical significance [40].

### Interim analysis

An interim analysis will be conducted after the enrollment of 25 patients in each group. This analysis is intended solely for exploratory purposes and will focus on estimating effect sizes. No formal stopping rules are defined.

### Study quality control

All measurement instruments used in this trial have been examined and demonstrate excellent validity and reliability [30–32, 38]. As university hospital, UKGM is best qualified in conducting research. All investigators have been trained in the assessment tools and interventions being evaluated.

## Discussion

This protocol presents a pilot prospective single-blinded controlled study designed to assess further risks of dysphagia following TEE. The trial aims to address several outcomes which will be correlated with the severity of dysphagia after TEE, building on the findings from the initial trial.

The initial TEDRAS study revealed a notable increase in all dysphagia outcome scores in the intervention group immediately following TEE, alongside a significant increase in residue scores for all consistencies in the valleculae, and for small and large liquid boluses in the piriform sinus. These findings align with Grimm et al. [41], who identified residue as the most common dysphagia symptom in patients post-intraoperative TEE. Additionally, a prospective randomized trial by Chin et al. [24] confirmed that prolonged intraoperative TEE placement in the esophagus negatively impacts swallowing in cardiac patients. However, the initial TEDRAS trial did not evaluate the effect of TEE duration on swallowing. Based on the TEE probe’s diameter, weight and the anatomical proportions of the human oral cavity and tongue, it was hypothesized in the initial trial that the probe exerts excessive pressure on the oral, pharyngeal and laryngeal tissues, impairing the motility of the tongue, tongue base, pharyngeal wall, larynx and upper esophageal sphincter. Lastly, the initial TEDRAS study did not investigate the influence of anaesthesia on swallowing. The aim of the TEDRAS 2 follow-pp study is to address the above-mentioned limitations of the initial TEDRAS study in order to determine which factors are leading to dysphagic symptoms after TEE and thus to ensure a good aspiration prevention for stroke patients undergoing TEE.

### Oversight and monitoring

#### Trial management group

The trial management group (TMG) of the coordinating centre, Department of Neurology of the UKGM, includes the PI, key clinical investigators (MDs and SLTs as well as members of nurse staff) and non-clinical investigators (study nurse). The PI from the Department of Neurology at UKGM monitors data collection, data confidentiality, adverse events reporting and data analysis. Any adverse or unexpected events wil be promptly reported to the research project lead and to the Ethics Committee of the JLU. The trial´s dialy operations and management are overseen by the responsible MD and SLT from the Department of Neurology at UKGM. The MDs form the Department of Cardiology are responsible for the trial related conduct of TEE and report of any relevant trial related events to the PI. The TMG meetings are scheduled four times annually.

#### Trial steering committee

The Trial Steering Committee (TSC) includes independent medical experts in stroke management and a cardiologist acting as chairpersons and a statistician. The main task of TSC is to offer an independent oversight of the trial as a whole and to offer guidance through the independent chairperson. The TSC bears the final responsibility whether to continue the trial at any point of time.

#### Risks, burdens and benefits

Both interventions, FEES and TEE, are conducted according to national and international guidelines [42–46]. The additional FEES may improve the detection and, if necessary, treatment of dysphagia. Furthermore, the results of the study may help to improve the diagnosis and treatment of swallowing disorders in other patients in the future.

#### Modification and inquiry

Any modification of the study protocol will be submitted for amendment to the Ethics Board of JLU.

#### Confidentialty

All personal data recorded in the consent form and case report form will be anonymized and pseudonomized and saved in a secured database. Monitoring of research data will be conducted by the PI and may only be accessed by authorized members of the trial team.

#### Dissemination policy

The findings of the trial will be disseminated to peer-reviewed scientific journals and presented at scientific conferences.

### Trial status

This protocol is presented in #1 version, 01.12.2024. The patient recruitment has started in December 2024. Trial completion is planned for January 2026. 

## Data Availability

The datasets created and/or analyzed in this study are not publicly accessible but can be obtained from the corresponding author upon reasonable request.

## Abbreviations

FEES: Flexible Endoscopic Evaluation of Swallowing
TEE: Transoesophageal echocardiography
TTE: Transthoracal echocardiography
SSRS: Secretion Severity Rating Scale
YSv: Yale Pharyngeal Residue Severity Rating Scale for Valleculae
YSps: Yale Pharyngeal Residue Severity Rating Scale for Piriform Sinus
PAS: Penetration-Aspiration-Scale
FOIS-G: Function Oral Intake Scale (German Version)
NIHSS: National Institutes of Health Stroke Scale
MRS: Modified Rankin Scale
BI: Barthel Index
CCT: Cranial computed tomography
MRI: Magnetic resonance imaging
PI: Prinicipal investigator
UKGM: University Hospital Giessen and Marburg
MD: Medical Doctor
SLT: Speech and language therapist
